# Supplementary material for: Myostatin (MSTN) Gene Indel Variation and Its Associations with Body Traits in Shaanbei White Cashmere Goat
Source: Animals (Basel). 2020 Jan 19;10(1):168. doi: 10.3390/ani10010168 (PMC7022945; doi:10.3390/ani10010168)
Supplement: Supplementary file 1 [file animals-10-00168-s001.pdf]

Table S1. Genetic parameters of the 5 bp indel within the *GDF9* gene in different goat breeds.

Breeds	Sample Sizes	Frequencies					Homo	Hetero	Ne	PIC	HWE *p*-Values	Reference
		Genotypes			Alleles							
		DD	ID	II	D	I						
Black Bengal	n = 160	0.981	0.019	0	0.990	0.010	0.981	0.019	1.019	0.018	0.009	Singh et al., 2014
Sirohi	n = 24	0.875	0.125	0	0.938	0.062	0.883	0.117	1.133	1.133	0.063	Singh et al., 2014
Osmanabadi	n = 23	0.956	0.044	0	0.978	0.022	0.958	0.042	1.044	0.042	0.022	Singh et al., 2014
Jakhrana	n = 21	1.000	0	0	1.000	0	1.000	0	0	0	0	Singh et al., 2014
Jamunapari	n = 24	0.916	0.084	0	0.958	0.042	0.920	0.080	1.087	0.077	0.042	Singh et al., 2014
Barbari	n = 28	0.893	0.107	0	0.946	0.054	0.899	0.101	1.113	0.096	0.054	Singh et al., 2014
Marwari	n = 10	0.800	0.200	0	0.900	0.100	0.820	0.180	1.220	0.164	0.100	Singh et al., 2014
Markhoz	n = 150	0.850	0.150	0	0.920	0.080	0.853	0.147	1.173	0.136	0.309	Khani et al., 2017
Boer	n = 482	0.490	0.430	0.080	0.700	0.300	0.580	0.420	1.724	0.332	0	Zhang et al., 2012
Maton	n = 94	0.820	0.160	0.020	0.900	0.100	0.820	0.180	1.220	0.164	0.401	Zhang et al., 2012
Haimen	n = 45	0.490	0.420	0.090	0.700	0.300	0.580	0.420	1.724	0.332	0.073	Zhang et al., 2012
Nubi	n = 66	0.210	0.550	0.240	0.490	0.510	0.500	0.500	1.999	0.375	0.002	Zhang et al., 2012
Backcrossed offspring	n = 135	0.594	0.354	0.052	0.771	0.229	0.647	0.353	1.546	0.291	0.257	Li et al., 2008

Note: HWE: Hardy–Weinberg equilibrium; Homo: Homozygosity; Hetero: Heterozygosity; Ne: Effective allele numbers; PIC: Polymorphism information content. Backcrossed offspring: Boer Goat backcrossed offspring to Tangshan Dairy Goat. Genetic parameters include genotypes and alleles are as reported in previous researches. Others are calculated in this study.

Table S2. Relationship between the 5 bp indel locus within the *MSTN* gene and the growth traits in SBWC adults (LSM [a] ± SE) ($p < 0.05$).

Growth Traits	Observed Genotypes (LSM [a] ± SE)		*p*-Values
	DD	ID	
Body Height (cm)	55.06 ± 0.22 (n = 403)	54.88 ± 0.57 (n = 64)	0.75
Height at Hip Cross (cm)	58.57 ± 0.23 (n = 401)	58.07 ± 0.60 (n = 64)	0.42
Body Length (cm)	67.99 ± 0.28 (n = 403)	68.39 ± 0.72 (n = 64)	0.60
Hip Width (cm)	15.26 ± 0.10 (n = 406)	15.11 ± 0.25 (n = 64)	0.58
Chest Width (cm)	20.96 ± 0.19 (n = 404)	20.64 ± 0.40 (n = 64)	0.53
Cannon Circumference (cm)	8.32 ± 0.04 (n = 406)	8.16 ± 0.09 (n = 64)	0.09
Chest Depth (cm)	29.36 ± 0.16 (n = 404)	29.07 ± 0.37 (n = 64)	0.50
Body Weight (kg)	42.64 ± 0.56 (n = 386)	42.54 ± 1.50 (n = 58)	0.95
Heart Girth (cm)	84.00 ± 0.44 (n = 405)	82.81 ± 0.99 (n = 64)	0.31
Body Trunk index	122.11 ± 0.93 (n = 408)	121.26 ± 1.08 (n = 64)	0.73
Body Length index	122.33 ± 0.97 (n = 408)	125.36 ± 1.08 (n = 64)	0.23
Heart Girth index	151.20 ± 1.31 (n = 408)	151.61 ± 2.15 (n = 64)	0.90
Cannon Circumference index	14.99 ± 0.13 (n = 408)	14.96 ± 0.21 (n = 64)	0.92
Chest Width index	70.61 ± 0.60 (n = 408)	70.99 ± 1.00 (n = 64)	0.81
Hip Width index	136.51 ± 1.41 (n = 408)	137.16 ± 2.11 (n = 64)	0.86

Table S3. Correlation matrix of different growth traits in SBWC.

Kid	BH	HHC	CC	BW	Adult	BH	HHC	CC	BW
BH		2.50×10^{-17}	3.8×10^{-2}	1.24×10^{-3}	BH		7.3×10^{-77}	0.1×10^{-2}	8.08×10^{-15}
HHC	0.83 **		8.59×10^{-8}	7.49×10^{-8}	HHC	0.70 **		3.65×10^{-12}	6.18×10^{-36}
CC	0.08 **	0.20 **		5.09×10^{-4}	CC	0.15 **	0.30 **		6.74×10^{-31}
BW	0.33 **	0.52 **	0.35 **		BW	0.34 **	0.53 **	0.49 **	

Note: *P*-values and correlation index for correlation matrix of different growth traits were shown by the upper and lower triangles in the table, respectively. BH: Body height (n = 84); HHC: Height at hip cross (n = 602); CC: Cannon bone circumference (n = 602); BW: Body weight (n = 602); Cells with ** differed significantly ($p < 0.01$).
